# RNA polymerase II: the elephant in the room

**DOI:** 10.1016/j.tig.2026.01.008

**Published:** 2026-03-11

**Authors:** Steven Henikoff, Steven Hahn

**Affiliations:** 1 Basic Sciences Division, Fred Hutchinson Cancer Center, Seattle, WA 98109, USA; 2 Howard Hughes Medical Institute, Chevy Chase, MD 20815, USA

## Abstract

How genes respond to external signals is poorly understood, with transcription factors and histones proposed as mediators of signals to RNA polymerase II (Pol II). However, recent work shows that Pol II is the direct target of more than 100 protein kinases, a previously underappreciated mechanism for generating epigenetic complexity.

## Phosphorylation of the carboxy-terminal domain regulates RNA polymerase II activity

The central dogma of molecular biology, in which information flows from DNA to RNA to protein, depends on choosing which genes to express in a cell. However, how this choice is encoded and transmitted to eukaryotic RNA polymerase II (Pol II) is not fully understood. Transcription factor (TF) binding can lead to Pol II recruitment, but TFs do not directly bind to Pol II or to the machineries responsible for DNA melting, pausing, and release into productive elongation [1]. Rather, the unstructured activation domains of TFs function, in part, to promote phosphorylation of the 52 tandem copies of a Tyr^1^–Ser^2^–Pro^3^–Thr^4^–Ser^5^–Pro^6^–Ser^7^ (YSPTSPS) heptapeptide repeat comprising the carboxy-terminal domain (CTD) of the largest subunit [2]. Specifically, the CTD is phosphorylated by transcriptional cyclin-dependent kinases (CDKs) on Ser^5^ during initiation and on Ser^2^ during elongation. However, exactly how activation of transcription is achieved and how information from outside the cell reaches Pol II has remained mysterious, despite decades of study.

A popular hypothesis is that signals are transmitted via histone modifications to dictate downstream events [3]. However, the key histone-modifying enzymes that are associated with active transcription are themselves targeted to their lysine and arginine substrates on nucleosomes by Pol II: Histone H3K4 is mono-, di-, and trimethylated by the Set1 complex, which associates with the Ser^5^-phosphorylated CTD close to the promoter, while Set2-family enzymes associate with Ser^2^-phosphorylated CTD farther downstream [4]. This dependence on the CTD phosphorylation state of histone-modifying enzymes is consistent with them being ‘cogs’ in the regulation of transcription, working together with other nonhistone chromatin proteins and ATP-dependent nucleosome remodelers.

Although regulation by CDK-catalyzed modification of Ser^2^ and Ser^5^ has been called the ‘CTD code,’ these modifications promote Pol II activity at different stages in the transcription cycle and, therefore, do not constitute a combinatorial code akin to the Morse code or the genetic code. With relatively few studies of phosphorylation of the three other hydroxyl amino acids on the CTD (Tyr^1^, Thr^4^, and Ser^7^), there has been scant evidence for combinatorial complexity, such as has been proposed for the ‘histone code.’ Furthermore, phosphorylation of these CTD ‘orphan’ residues was not thought to be important for regulating Pol II activity, except in specific contexts [5]. However, interference with antibody binding to these residues by abundant Ser^2^ and Ser^5^ phosphates in close proximity on the unstructured CTD peptide may have obscured detection.

## Revisiting the CTD code

A recent study of receptor kinases has now brought the CTD code back into the limelight, presenting a new paradigm that may explain how extracellular signals regulate Pol II activity. Using two different assays on a collection of 427 purified human kinases, Dabas *et al.* [6] identified 117 purified human kinases that transferred phosphates to an unmodified (YSPTSPS)_14_ peptide in both assays. Their assays identified not only known transcriptional CDKs responsible for Ser^2^ and Ser^5^ phosphorylation but also well-studied members of various serine/threonine kinase (STK) and tyrosine kinase (TK) families from all major branches of the human kinase evolutionary tree. In total, approximately 80% of the kinases in their sample were specific for a particular CTD amino acid, as they failed to modify mutated 14mer peptides lacking the targeted residue. Among the 54 Tyr^1^-specific kinases that the screen identified, many receptor kinases were more active than nonreceptor kinases, consistent with the possibility that these and other receptor kinases that bind ligands on the cell surface function to transmit signals from the environment directly to Pol II.

To test this interpretation, the authors focused on two well-studied receptor TKs: insulin and epidermal growth factor (EGF) receptors. In both cases, they confirmed CTD-specific activity *in vitro* and showed TF-dependent nuclear colocalization with Pol II *in vivo*. In the case of the EGF receptor, nuclear localization was observed cytologically within 2 min after the addition of the EGF ligand, and binding to target early responder gene promoters and CTD-Tyr^1^ phosphorylation began within 5 min, followed by the appearance of mRNA. This provides compelling evidence that receptor TKs are guided by TFs to directly target the Pol II CTD and activate transcription. As 282 of the 427 purified kinases in the screen phosphorylated the CTD in at least one of the two assays, it appears that a large fraction, perhaps most, of the protein kinases in the human genome target the Pol II CTD.

## Mechanistic implications

**Figure 1.**
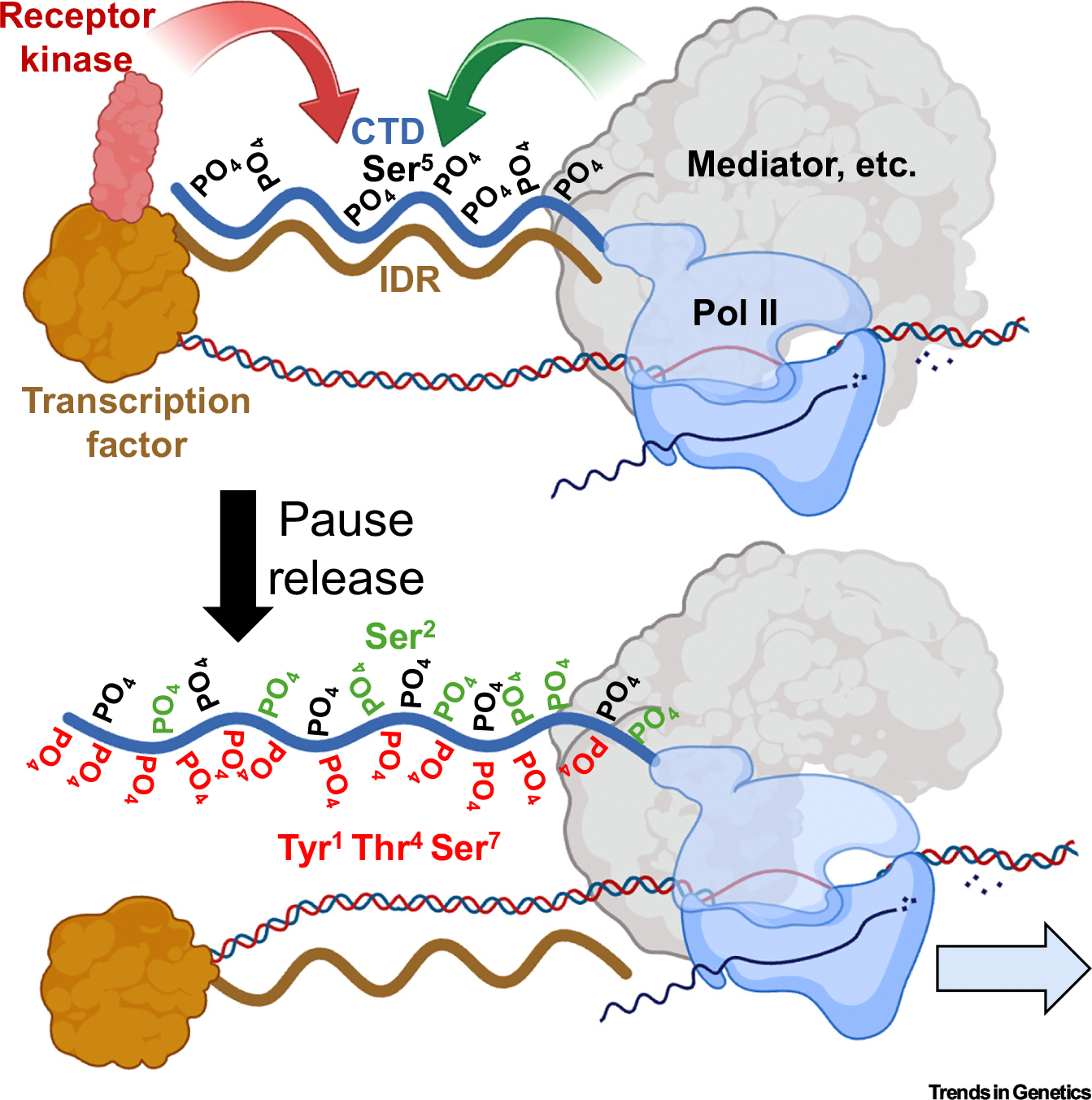
Model for Pol II CTD regulation by receptor kinases. Top: At a promoter, the CTD becomes Ser^5^-phosphorylated (black PO_4_) and pauses before productive elongation. The IDR of a sequence-specific TF nearby undergoes weak binding interactions with the CTD and a receptor kinase bound to the TF then phosphorylates the CTD on Tyr^1^, Thr^4^, or Ser^7^ (left arrow), while transcriptional CDKs phosphorylate Ser^2^ (right arrow). Bottom: At physiological pH, each phosphate contributes approximately two negative charges to the CTD each centered on the phosphate, and the charge repulsion and bulk of the additional phosphates (red PO_4_ from the receptor kinase and green PO_4_ from transcriptional CDKs) extend the CTD and release Pol II from the TF IDR, the Mediator complex, and elongation factors DRB sensitive-inducing factor (DSIF) and Negative elongation factor (NELF). CDKs: cyclin-dependent kinases; CTD: carboxy-terminal domain; IDR: intrinsically disordered region; Pol II: RNA polymerase II; TF: transcription factor. Created in BioRender (https://BioRender.com/v439hm8).

The CTD is an example of an intrinsically disordered region (IDR), participating in homotypic interactions to form condensates in its unmodified state and in heterotypic interactions with IDRs of TFs [7]. IDRs can contribute to sequence specificity [8,9], and weak IDR heterotypic interactions that stabilize TF binding have been confirmed by live-cell imaging [10,11]. These and other such observations raise the possibility that weak CTD–TF heterotypic interactions, which stabilize TF binding, are disrupted by phosphorylation, which at physiological pH contributes approximately two negative charges per phosphate to the CTD [12] (Figure 1 top). The association of a protein kinase with a TF bound near the promoter and the phosphorylation of the CTD increase its net charge, which, together with Ser^2^ phosphorylation by transcriptional CDKs, might exceed the threshold for pause release. We propose that the distributed charge and bulk of so many additional phosphates disrupt the weak heterotypic interactions between TF IDRs and the CTD, and charge repulsion extends the CTD [5,13] (Figure 1 bottom). In support of this interpretation, CTD phosphorylation disrupts condensates *in vivo* [7] and reverses droplet formation *in vitro* [13]. A signal integration rheostat based on net negative charge would have essentially unlimited intermediate charge states for fine-tuning pause release, leading to productive elongation.

Other enzymes known to modify the CTD include peptidyl-prolyl cis/trans isomerases, which can alter CTD conformation and affect kinase accessibility by targeting either of the two Ser-Pro motifs in the heptapeptide [13]. Thus, all seven amino acids in the CTD are subject to enzymatic modification and nearest-neighbor effects [12]. Autophosphorylation of receptor kinases upon ligand binding and CTD phosphorylation may also provide potential docking sites to further increase regulatory flexibility.

## Perspective

Deciphering CTD phosphorylation complexity in different cell types and under different environmental conditions will require technological advances in epigenomics, proteomics, and imaging technologies. Yeast is ideal for mechanistic studies of TF-mediated CTD phosphorylation, with well-understood TF signaling networks and relatively simple promoter architectures. Indeed, recent work has shown that yeast promoter TF binding often does not predict the gene that is activated by binding, suggesting an intermediate layer of regulation [14], and it will be interesting to investigate whether phosphorylation and/or prolyl cis/trans isomerization of the CTD is involved in yeast responses to environmental signals.

The approximately 250–300 amino acid protein kinase catalytic domain is ancient: STKs are found in archaea, bacteria, and eukaryotes, and TKs diverged from the STKs prior to the origin of eukaryotes. Both lineages have diversified enormously for specific cellular functions [15]. It would, therefore, appear that STKs and TKs have retained their simple ancestral function, phosphorylating the Pol II CTD, while diversifying over long evolutionary periods to ‘moonlight’ for the regulation of a wide variety of cellular protein substrates. As both receptor and transcriptional kinases have been among the most important biomarkers and therapeutic targets for cancer and other diseases, a concerted effort centered on this new paradigm of transcriptional regulation should be a top priority for biomedical research.
